# Characteristics of dry eye patients with thick tear film lipid layers evaluated by a LipiView II interferometer

**DOI:** 10.1007/s00417-020-05044-5

**Published:** 2021-01-06

**Authors:** Yunjin Lee, Joon Young Hyon, Hyun Sun Jeon

**Affiliations:** 1 Department of Ophthalmology, Seoul National University Bundang Hospital, Seongnam, South Korea; 2 Department of Ophthalmology, Seoul National University College of Medicine, Seoul, South Korea

**Keywords:** Dry eye disease, Interferometry, Lipid layer thickness, LipiView

## Abstract

**Purpose:**

To investigate the characteristics of eyes with dry eye disease (DED) whose lipid layer thickness (LLT) measured 100 nm on a LipiView II interferometer and compare the DED parameters of them to those with LLT below 100 nm.

**Methods:**

A total of 201 eyes of 102 enrolled DED patients (mean age 56.4 ± 11.8 years) were classified into 3 groups according to their average LLT; < 60 nm as thin-LLT (*n* = 49), 60–99 nm as normal-LLT (*n* = 77), and 100 nm as thick-LLT (*n* = 75). LLT, meiboscore, Schirmer *I* test, tear film break-up time (TBUT), ocular surface staining (OSS), and ocular surface disease index (OSDI) were assessed.

**Results:**

The OSS and TBUT were significantly worse in the thick-LLT group than in the normal-LLT group (*p* = 0.020, and *p* = 0.028, respectively). The OSDI was significantly higher in the thick-LLT group than in the thin-LLT group (*p* = 0.006). However, the meiboscore was not different among the three groups (*p* = 0.33). Age, OSS, and OSDI showed a positive correlation with LLT (*r* = 0.16, *p* = 0.023; *r* = 0.213, *p* = 0.003; and *r* = 0.338, *p* = 0.001, respectively). In sensitivity analyses, eyes with corneal erosions had a significantly higher average LLT (*p* = 0.015), higher OSDI (*p* = 0.009), shorter TBUT (*p* < 0.001), and shorter Schirmer *I* value (*p* = 0.024) than those with clear corneas.

**Conclusion:**

The average LLT of eyes with corneal erosions was thicker than those without erosions, suggesting that the LLT of 100 nm in the eyes with corneal erosions should not be regarded as a stable physiologic condition. Cautious interpretation of LLT along with other dry eye parameters is required.

**Supplementary Information:**

The online version contains supplementary material available at 10.1007/s00417-020-05044-5.

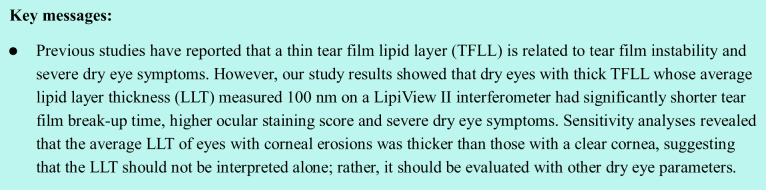

## Introduction

The classic model of the tear film originated in the 1950s and separated the tear film into three layers: the glycocalyx (mucin) layer, the intermediate aqueous layer, and the outermost tear film lipid layer (TFLL) [1]. The outermost TFLL is formed almost exclusively from lipids and attached and/or intercalated proteins. It is a two-layered structure consisting of an inner polar layer and an outer nonpolar lipid layer that is in contact with the air [1, 2]. The meibomian glands secrete meibum which is the main source of lipids in the TFLL [3]. Meibum plays a critical role in tear film stability and prevents tear evaporation from the aqueous tear film layer [4]. A previous study reported that impaired or absent TFLL disturbs the stability of the tear film and in these circumstances, tear evaporation increases fourfold [5]. Blackie et al. found that 3 of 4 patients reporting severe dry eye symptoms had relatively thin lipid layers (less than 60 nm), while Finis et al. suggested that a lipid layer thickness (LLT) of less than or equal to 75 nm could be used to detect obstructive meibomian gland dysfunction (MGD) with a sensitivity of 65.8% and a specificity of 63.4% [6, 7]. However, unlike previous reports, we have occasionally treated patients with severe dry eye symptoms who present with a normal or even thick LLT on clinical examination. Furthermore, there is a paucity of data that evaluates the characteristics of dry eye patients with thicker LLT values.

The LipiView II interferometer (TearScience Inc., Morrisville, NC, USA) is the first clinically available instrument to allow automated measurement of the LLT of the tear film with nanometer accuracy by analyzing the interferometric pattern of the tear film [7]. The LipiView II is limited in its analysis of LLT of more than 100 nm, since it can only show an upper cut-off LTT value of 100 nm. Considering our previous experience with thick-LTT dry eye patients, the limited data on such cases, and the upper cut-off value of the LipiView II interferometer, we aimed to investigate the characteristics of patients with dry eye disease (DED) whose LLT measured 100 nm on a LipiView II interferometer and compare their DED characteristics with those of patients with LLT values of < 100 nm.

## Material and methods

### Participants

We performed a retrospective analysis of 102 patients who had been diagnosed with DED at the Seoul National University Bundang Hospital between September 2016 and November 2017. Of the 204 eyes obtained from 102 patients, 3 eyes failed to get measured for LLT; thus, 201 eyes were enrolled in this study. The study was approved by the institutional review board (IRB) of Seoul National University Bundang Hospital (IRB identification number: B-2003-601-106) and was conducted as per the tenets of the Declaration of Helsinki. The requirement of informed consent was waived by the review board.

The inclusion criteria were as follows: (1) patient age of 20 years or older; (2) presence of DED assessed by the Dry Eye Workshop [8]; (3) eyes whose LLT was evaluated with the LipiView II interferometer. According to the average LLT, eyes were classified into one of three groups: thin-LLT (< 60 nm), normal-LLT (60–99 nm), and thick-LLT (100 nm). The exclusion criteria were as follows: the presence of any uncontrolled systemic disease, presence of any severe ocular surface disease and/or corneal epithelial pathology, previous ocular trauma or surgery other than uncomplicated cataract surgery, history of cataract surgery within the last 6 months, dysfunctions or structural abnormalities of the eyelid (e.g., incomplete closure, nasolacrimal obstruction), contact lens wear, and continuous eye drop use (e.g., antibiotics, antivirals, steroids, glaucoma medications, lipid emulsion eyedrops, and lubricant ointment) other than artificial tears that do not contain lipids.

### Lipid layer thickness and meiboscore

The measurement of LLT was achieved by the LipiView II interferometer, as previously described [6]. In brief, the LipiView II interferometer records a 20-s video of the tear film interference pattern and subsequently displayed data in interferometric color units (ICU), where 1 ICU reflects approximately 1 nm of LLT. The maximum, minimum, and average LLT from all frame averages were obtained. Data were obtained for each study eye. The meibomian glands in the lower eyelids were also evaluated with LipiView II. Partial or complete loss of meibomian glands was scored as meiboscore from 0 to 3 for each eyelid, where 0: no gland dropout; 1: < 33%; 2: 33–67% loss; and 3: > 67% dropout [9].

### Other dry eye parameters

The following tests were conducted to evaluate the dry eye characteristics: Schirmer *I* test, tear film break-up time (TBUT), corneal and conjunctival staining using Sjögren’s International Collaboration Clinical Alliance (SICCA) ocular surface staining (OSS) method, and a symptom questionnaire—the ocular surface disease index (OSDI). All the tests were performed by an experienced cornea specialist without knowledge of the results of the LLT and meiboscore.

The Schirmer *I* test was performed without the instillation of topical anesthetics, by placing a pre-calibrated dry filter strip (ColorBar; EagleVision Inc., Memphis, TN) on the mid-lateral portion of the lower fornix. The strips were removed after 5 min, and the amount of wetting was recorded. The patients were asked to close their eyes during the test.

TBUT was measured by placing a drop of sterile saline on a fluorescein sodium-impregnated paper strip (Haag-Streit International Con., Ltd, Koeniz, Switzerland). Patients were asked to blink for a few seconds, and the time before the first defect appeared on the stained tear film was measured at the slit lamp using the cobalt blue filter.

OSS was recorded using fluorescein in the same way as previously described [10]. Corneal punctate epithelial erosions (PEEs) were graded as 0 (no PEE), 1 (1–5 PEEs), 2 (6–30 PEEs), or 3 (> 30 PEEs). An additional point was added if (1) PEE occurred in the central 4 mm diameter portion of the cornea; (2) 1 or more filaments were seen anywhere on the cornea; or (3) 1 or more patches of confluent staining, including linear stains were found anywhere on the cornea. Conjunctival epithelial erosions were graded as 0 (0 to 9 dots on the interpalpebral bulbar conjunctiva), 1 (10–32 dots), 2 (33–100 dots), and 3 (> 100 dots). The nasal and temporal bulbar conjunctivae were graded separately.

Subjective symptoms were graded on a numerical scale from 0 to 4, according to the validated 12-item OSDI questionnaire. The total OSDI was calculated using the following formula: OSDI = (sum of scores for all questions answered × 100)/(total number of answered questions × 4). The values ranged from 0 to 100.

### Statistical analysis

Statistical analyses were performed using the SPSS for Windows software (version 23.0; SPSS Inc., Chicago, IL, USA). Since a majority of the variables did not have a normal distribution, nonparametric tests were adopted. The analyses included the frequency for categorical data and the median (range) for continuous data. Fisher’s exact test was used to compare categorical variables, and the Kruskal-Wallis test was used to compare the groups for numeric variables. Linear regression analyses were computed to evaluate the impact of clinical variables on LLT. Pearson’s correlation coefficients were calculated to explore the relationships between variables. A *p* value of < 0.05 was considered statistically significant.

## Results

### Characteristic of eyes classified into three groups according to average LLT

Of the total 102 DED patients, 88 were female, and 14 were male. Of the total 201 eyes, 49 eyes were classified into the thin-LLT group, 77 eyes in the normal-LLT group, and 75 eyes in the thick-LLT group. Table 1 represents the characteristics of all 3 groups. The mean ± SD of the average LLT was 45.18 ± 9.63 nm, 77.09 ± 10.94 nm, and 100 nm in the thin-LLT group, normal-LLT group, and thick-LLT group, respectively (*p* < 0.001).

**Table 1:** Characteristics of eyes with dry eye disease classified according to lipid layer thickness

	Total (*n* = 201)	Thin-LLT < 60 (*n* = 49)	Normal-LLT ≥ 60, < 100 (*n* = 77)	Thick-LLT = 100 (*n* = 75)	*p* value^*^
Age, years (range)	56.38 ± 11.78 (23–84)	49.61 ± 12.65 (23–78)	59.36 ± 10.99 (34–84)	57.75 ± 10.27 (37–83)	< 0.001
Sex, *n* (%)					0.04^#^
Male	28 (13.9)	11 (22.4)	12 (15.6)	5 (6.7)	
Female	173 (86.1)	38 (77.6)	65 (84.4)	70 (93.3)	
LLT, nm					
Average	77.86 ± 22.66	45.18 ± 9.63	77.09 ± 10.94	100	< 0.001
Minimum	60.82 ± 24.95	33.29 ± 8.96	55.61 ± 15.92	84.16 ± 16.98	< 0.001
Maximum	88.27 ± 16.80	64.69 ± 14.91	91.86 ± 9.74	100	< 0.001
Differentiation	27.28 ± 17.91	31.12 ± 31.12	34.32 ± 34.32	17.53 ± 17.53	< 0.001
Meiboscore	1.45 ± 0.69	1.41 ± 0.84	1.39 ± 0.57	1.55 ± 0.70	0.330
Schirmer *I*, mm	10.93 ± 7.49	10.44 ± 7.20	11.56 ± 7.20	10.59 ± 8.64	0.72
TBUT, s	2.22 ± 1.32	2.43 ± 1.63	2.43 ± 1.31	1.87 ± 1.04	0.028
OSS	1.23 ± 1.85	0.94 ± 1.79	0.96 ± 1.63	1.71 ± 2.02	0.020
OSDI	52.13 ± 21.77	44.14 ± 24.56	48.82 ± 19.55	61.15 ± 18.23	0.006

*LLT*, lipid layer thickness; *TBUT*, tear film break-up time; OSS, ocular staining score; *OSDI*, ocular surface disease index

^*^Analysis of variance (ANOVA)

^#^Chi-square test

The mean patient age of the thin-LLT group (49.6 ± 12.7 years) was significantly younger than that of the normal-LLT (59.4 ± 10.9 years, *p* < 0.001, ANOVA, followed by Bonferroni post hoc test) and thick-LLT groups (57.8 ± 10.3 years, *p* < 0.001, ANOVA, followed by Bonferroni post hoc test). The proportion of female patients in the thick-LLT group (93.3%) was significantly higher than that of the thin-LLT groups (77.6%; *p* = 0.01, chi-square test), and it was also higher but not statistically significant compared with the normal-LLT (84.4%; *p* = 0.081, chi-square test).

The Schirmer *I* value was higher in the normal-LLT group (11.56 ± 7.20 mm) than in the thin-LLT (10.44 ± 7.20 mm; *p* = 0.99, ANOVA, followed by Bonferroni post hoc test) and thick-LLT groups (10.59 ± 8.64 mm; *p* = 0.99, ANOVA, followed by Bonferroni post hoc test). However, these differences were not statistically significant.

The TBUT was significantly shorter in the thick-LLT group (1.87 ± 1.04 s) than in the normal-LLT and thin-LLT groups (2.43 ± 1.31 s, 2.43 ± 1.63 s, respectively, *p* = 0.028, ANOVA), and the OSS was significantly worse in the thick-LLT group (1.71 ± 2.02) than in the normal-LLT or thin-LLT groups (0.96 ± 1.63, 0.94 ± 1.79, respectively, *p* = 0.020, ANOVA). The OSDI of the thick-LLT group (61.15 ± 18.23) was significantly higher than that of the normal-LLT or thin-LLT groups (48.82 ± 19.55, 44.14 ± 24.56, respectively, *p* = 0.006, ANOVA). However, the meiboscores were not different among the 3 groups (*p* = 0.33).

### Correlations between lipid layer thickness and other clinical parameters

The average LLT showed a significantly positive correlation with age, OSDI, and OSS (*r* = 0.16, *p* = 0.023; *r* = 0.338, *p* = 0.001; *r* = 0.213, *p* = 0.003, respectively) (Table 2). The Schirmer *I* test value, TBUT, and meiboscore showed no significant correlations with average LLT (*r* = − 0.018, *p* = 0.826; *r* = − 0.152, *p* = 0.051; *r* = 0.081, *p* = 0.252, respectively).

**Table 2:** Correlation analyses

		LLT	Age	OSDI	OSS	Schirmer *I*	TBUT	Meiboscore
LLT	*r*		0.160^*^	0.338^#^	0.213^#^	− 0.018	− 0.152	0.081
	*p*		0.023	0.001	0.003	0.826	0.051	0.252
	*n*		201	86	197	153	166	201
Age	*r*	0.160^*^		− 0.171	0.027	− 0.073	− 0.075	− 0.251^#^
	*p*	0.023		0.115	0.706	0.372	0.340	< 0.001
	*n*	201		86	197	153	166	201
OSDI	*r*	0.338^#^	− 0.171		0.258^*^	− 0.027	− 0.096	0.045
	*p*	0.001	0.115		0.016	0.803	0.414	0.683
	*n*	86	86		86	86	74	86
OSS	*r*	0.213^#^	0.027	0.258^*^		− 0.302^#^	− 0.466^#^	− 0.049
	*p*	0.003	0.706	0.015		< 0.001	< 0.001	0.490
	*n*	197	197	86		153	166	197
Schirmer *I*	*r*	− 0.018	− 0.073	− 0.027	− 0.302^#^		0.473^#^	− 0.117
	*p*	0.826	0.372	0.803	< 0.001		< 0.001	0.151
	*n*	153	153	86	153		130	153
TBUT	*r*	− 0.152	− 0.075	− 0.096	− 0.466^#^	0.473^#^		− 0.002
	*p*	0.051	0.340	0.414	< 0.001	< 0.001		0.977
	*n*	166	166	166	166	130		166
Meiboscore	*r*	0.081	0.251^#^	0.045	− 0.049	− 0.117	− 0.002	
	*p*	0.252	< 0.001	0.683	0.490	0.151	0.977	
	*n*	201	201	197	197	153	166	

*LLT*, lipid layer thickness; *TBUT*, tear film break-up time; *OSS*, ocular staining score; *OSDI*, ocular surface disease index

^*^Statistically significant at *p* < 0.05

^#^Statistically significant at *p* < 0.001

### Sensitivity analyses

As the OSS of the thick-LLT group was significantly higher than that of the normal- and thin-LLT groups, we conducted subgroup analysis between the group with corneal erosions (OSS > 0, mean OSS of 2.48 ± 1.95) and without erosion (OSS = 0; Table 3). The corneas-with-erosions group had a significantly higher value of average LLT (*p* = 0.015), higher OSDI (*p* = 0.009), shorter TBUT (*p* < 0.001), and shorter Schirmer *I* value (*p* = 0.024).

**Table 3:** Comparison of dry eye parameters between eyes with clear corneas and erosive corneas

	Clear cornea (*n* = 99)	Erosive cornea (*n* = 98)	*p* value
Age, years	56.07 ± 11.71	57.31 ± 11.10	0.448
Average LTT, nm	73.96 ± 23.69	81.80 ± 20.98	0.015
Schirmer *I*, mm	12.45 ± 7.95	9.62 ± 7.36	0.024
TBUT, s	2.67 ± 1.42	1.80 ± 1.05	< 0.001
Total OSS	0	2.48 ± 1.95	< 0.001
Meiboscore	1.47 ± 0.76	1.42 ± 0.625	0.571
OSDI	45.62 ± 23.62	57.79 ± 18.48	0.009

*LLT*, lipid layer thickness; *TBUT*, tear film break-up time; *OSS*, ocular staining score; *OSDI*, ocular surface disease index

## Discussion

In this study, we have evaluated the characteristics of eyes with LLT measured 100 nm by a LipiView II interferometer and compared DED parameters by grouping dry eyes with various average LLTs according to the known normal ranges [6]. Interestingly, our results showed that eyes in the thick-LLT group (LTT = 100 nm) had a significantly shorter TBUT, higher OSS, and higher OSDI as compared to those in the thin- and normal-LLT groups. These results contrast with previous studies that reported that thin-LLT values are related to tear film instability and severe DED symptoms [6, 7, 11]. Isreb et al. reported a significant positive correlation between TBUT and the LLT in DED patients, and Eom et al. also found the same result with respect to obstructive MGD [12, 13]. A possible explanation for the discrepancy between our findings and previous studies is that patients in the thick-LLT group in our study may have a higher severity of DED with a relatively high OSS. Subgroup analysis revealed that eyes with corneal erosion had significantly shorter TBUT and Schirmer *I* values and higher average LLT values. These sensitivity analyses also support this explanation. The heterogeneity of dry eye patients included in our study also may have contributed to these differences. Considering these results, the LLT should not be interpreted alone but should be evaluated with other dry eye parameters. In this respect, the LLT values, especially for eyes with corneal erosions, should be interpreted with caution and the observation of a 100 nm LLT with the LipiView II in these eyes should not be regarded as a stable physiologic condition.

The TFLL stabilizes the entire tear film and prevents tear evaporation from the aqueous layer [14]. The TFLL can be evaluated not only with its thickness but also with its structure and composition [15]. LLT measurement with the LipiView II interferometer, which is capable of quantifying LLT automatically, is expected to be beneficial for the assessment and classification of DED and MGD. However, the exact thickness of the lipid layer that helps prevent tear evaporation is still not known. To date, there is no standardized data for its clinical interpretation. Thus, there is the potential to misinterpret the functioning of the TFLL if evaluated only by its thickness. As seen in our study, the thick-LLT group displayed higher OSS than the thin- and normal-LLT groups. However, it is not clear whether the LLT in patients with higher OSS is actually thicker or if the LLT values are artificially high due to measurement error. Recently, Jung et al. reported that, after adjusting other confounding factors, OSS showed little association with LLT [16]. However, since their subjects’ average OSS was relatively lower than our study, further studies are required to confirm the effect of corneal erosions on the measurement of LLT. As the LipiView II interferometry measures LLT based on the interference pattern of the ocular surface, corneal erosions might affect the color or pattern of the interferometer, which in turn affects the LLT value. Therefore, even with a thick lipid layer, if the homeostasis of the ocular surface is not controlled, patients may feel discomfort from their tear film instability.

In this study, there were no significant differences in other DED parameters (Schirmer *I*, TBUT, OSS, and OSDI) except for age between the thin- and normal-LLT groups. The correlation analyses of DED patients with an average LLT of < 100 nm confirmed that only age was significantly correlated with LLT (Online Resource Table S1). These results are supported by Jung et al.’s study that showed age had a strong influence on LLT in both the normal and dry eye groups [16]. These results, together with our findings, suggest that age should be adjusted when developing a normalized database of average LLT values. Also, among patients with mild DED, where there is no difference in any other dry eye parameter, LLT cannot be explained other than by its relationship with age.

The correlation between tear production and meibomian gland function has been suggested in previous studies. A recent study proposed that deficiency of the lipid layer induced tear film instability and secretion of tear fluid might increase as a compensatory response [17]. In their study, Schirmer’s test was adopted for determining tear fluid secretion and meiboscore for meibomian gland abnormality. However, in our study, neither meiboscore nor average LLT showed a correlation with Schirmer *I* values, which does not support the compensatory response of the tear secretion. Our results that the meiboscore had little correlation with LLT and was not different among the three groups indicate that evaluation of the meibomian gland’s structure does not reflect its function. As further studies add to our understanding of LLT and the meiboscore in patients with DED or MGD, our grasp of the compensatory response of tear fluid secretion is expected to deepen.

The significantly shorter TBUT and higher OSDI in the thick-LLT group in our study are contrary to previous studies. As mentioned earlier, one possible explanation for this discrepancy was the characteristics of the enrolled patients with thick-LLT and higher OSS. Tear film stability is most commonly evaluated by measuring TBUT with fluorescein [18]. More severe ocular surface damage, indicated by higher OSS, may contribute to the tear film instability and severe symptom scores in the thick-LLT group.

In our study, the thick-LLT group had a significantly higher proportion of female patients than the thinner LLT groups. Using Tearscope, Guillon and Maissa demonstrated a significantly poorer quality of the TFLL and thinner LLT in women over the age of 45 [19]. However, Jung et al., in their regression analyses using the LipiView interferometer, reported that women had a greater LLT than men [16]. They suggested that contamination of the TFLL in older female patients may have affected LLT evaluation using the LipiView interferometer. They recommend that both LLT values and the quality of the TFLL need to be evaluated. While still controversial, it has been reported that circulating androgens are necessary for maintaining normal meibomian function with substantial evidence for the presence of both androgen and estrogen receptors in human meibomian glands [20–25]. According to a previous study that measured the causal level of human meibomian lipids by meibometry, levels were higher in men than women from puberty until the end of the sixties, suggesting a potential hormonal effect on meibomian secretion [26]. Likewise, further studies are needed to consider the quality or contamination of the TFLL and effect of sex on LLT.

There are several limitations to this study. First, as our sample included only DED patients, we could not present a normal range of LLT or a cut-off value of LLT as a diagnostic criterion for MGD or DED. Further studies using normal controls would be helpful in this regard. However, we designed our study such that we could identify the characteristics of DED according to its LLT distribution. Second, we could not consistently evaluate the expression of meibomian glands or change of the lid margin due to the retrospective nature of the study, which may have affected the interpretation of the results and made it difficult to determine the presence of MDG and its type (obstructive or hypersecretory). However, we evaluated meiboscores, which, as per a recent study, are regarded as an effective diagnostic tool for MGD [27]. Third, a repeated examination is required to reflect seasonal and diurnal variations in a patient’s LLT and ocular surface status, which was not performed in our study. Fourth, the LipiView II interferometer had an upper cut-off LLT measurement value of 100 nm, and eyes with an LLT value of more than 100 nm may have contributed to a bias in the correlation analyses between LLT and other DED parameters. Fifth, there may be limitations of measurements related to the instability of TFLL: it is not constant over its entire surface area and assumed that the lipid in the upper and lower reservoirs combines to some extent during lid closure [6, 20]. However, minimum, maximum, and average LLTs showed significant differences among the three groups, and an average LLT is usually adopted as a value representing the LLT [6, 7, 16, 28]. Finally, given that the sample size may not be large enough to detect significant differences among the three groups or correlation, further prospective studies with a large sample size are required to support our findings.

In conclusion, our results suggest that the LLT of dry eye patients should not be interpreted alone; rather, they should be evaluated with other dry eye parameters, including corneal surface status. Considering that the average LLT of eyes with corneal erosions was thicker than those with clear corneas, assessing DES using simple numerical values of LLT should be avoided. The finding of 100 nm with the LipiView II in eyes with corneal erosions should not be regarded as a stable physiologic condition, and cautious interpretation is required. To date, there is no standardized normal range of LLT values, and there is scope for this range being described in the future. Further, our results suggest that an internally normalized database based on age and sex should be developed for using the LLT value as a diagnostic parameter. Further research that considers the effects of corneal erosions or the composition of the TFLL on LLT measurements is required.

## Supplementary information

ESM 1 (DOCX 27 kb)

## Data Availability

The datasets used and/or analyzed during the current study are available from the corresponding author on reasonable request.
