# Supplementary material for: Characteristics of dry eye patients with thick tear film lipid layers evaluated by a LipiView II interferometer
Source: Graefes Arch Clin Exp Ophthalmol. 2021 Jan 6;259(5):1235–41. doi: 10.1007/s00417-020-05044-5 (PMC8102443; doi:10.1007/s00417-020-05044-5)
Supplement: Supplementary file 1 — (DOCX 27 kb) [file 417_2020_5044_MOESM1_ESM.docx]

**Table S1. Correlation analyses average LLT below 100 nm**

|  |  | LLT | Age | OSDI | OSS | Schirmer I | TBUT | Meiboscore |
| --- | --- | --- | --- | --- | --- | --- | --- | --- |
| LLT^†^ | r |  | -0.020 | -0.036 | 0.013 | -0.087 | 0.305* | -0.025 |
|  | p |  | 0.895 | 0.722 | 0.906 | 0.427 | 0.002 | 0.809 |
|  | N |  | 46 | 98 | 82 | 85 | 98 | 98 |
| Age | r | -0.020 |  | 0.064 | 0.000 | 0.040 | 0.213 | 0.232 |
|  | p | 0.895 |  | 0.671 | 0.999 | 0.794 | 0.155 | 0.121 |
|  | N | 46 |  | 46 | 46 | 45 | 46 | 46 |
| OSDI^\|\|^ | r | -0.036 | 0.064 |  | -0.365* | -0.503* | -0.039 | 0.191 |
|  | p | 0.722 | 0.671 |  | 0.001 | <0.001 | 0.700 | 0.060 |
|  | N | 98 | 46 |  | 82 | 85 | 98 | 98 |
| OSS^§^ | r | 0.013 | <0.001 | -0.365* |  | 0.589* | -0.115 | -0.035 |
|  | p | 0.906 | 0.999 | 0.001 |  | <0.001 | 0.302 | 0.757 |
|  | N | 82 | 46 | 82 |  | 77 | 82 | 82 |
| Schirmer I | r | -0.087 | 0.040 | -0.503* | 0.589* |  | -0.077 | -0.147 |
|  | p | 0.427 | 0.794 | <0.001 | <0.001 |  | 0.484 | 0.179 |
|  | N | 85 | 45 | 85 | 77 |  | 85 | 85 |
| TBUT^‡^ | r | 0.305* | 0.213 | -0.039 | -0.115 | -0.077 |  | 0.024 |
|  | p | 0.002 | 0.155 | 0.700 | 0.302 | 0.484 |  | 0.818 |
|  | N | 98 | 46 | 98 | 82 | 85 |  | 98 |
| Meiboscore | r | -0.025 | 0.232 | 0.191 | -0.035 | -0.147 | 0.024 |  |
|  | p | 0.809 | 0.121 | 0.060 | 0.757 | 0.179 | 0.818 |  |
|  | N | 98 | 46 | 98 | 82 | 85 | 98 |  |

^†^LLT= lipid layer thickness; ^‡^TBUT = tear film break-up time; ^||^OSDI = ocular surface disease index; §OSS = ocular staining score

* P < 0.01 as a statistically significant
